# Mental health literacy and its associated factors among traditional healers toward mental illness in Northeast, Ethiopia: A mixed approach study

**DOI:** 10.1371/journal.pone.0298406

**Published:** 2024-02-23

**Authors:** Tamrat Anbesaw, Amare Asmamaw, Kidist Adamu, Million Tsegaw

**Affiliations:** 1 Department of Psychiatry, College of Medicine and Health Science, Wollo University, Dessie, Ethiopia; 2 Department of Health Service Management, College of Medicine and Health Science, Wollo University, Dessie, Ethiopia; Wolkite University, ETHIOPIA

## Abstract

**Background:**

Currently, the biggest issue facing the entire world is mental health. According to the Ethiopian Ministry of Health, nearly one-fourth of the community is experiencing any of the mental illness categories. Most of the cases were treated in religious and traditional institutions, which the community most liked to be treated. However, there were very limited studies conducted to show the level of mental health literacy among traditional healers.

**Aims:**

The study aimed to assess the level of mental health literacy and its associated factors among traditional healers toward mental illness found in Northeast, Ethiopia from September 1-30/2022.

**Method:**

A mixed approach cross-sectional study design was carried out on September 130, 2022, using simple random sampling with a total sample of 343. Pretested, structured questionnaires and face-to-face interviews were utilized for data collection. The level of Mental Health Literacy (MHL) was assessed using the 35 mental health literacy (35-MHLQ) scale. The semi-structured checklist was used for the in-depth interview and the FGD for the qualitative part. Data was entered using Epi-data version 4.6 and, then exported to SPSS version 26 for analysis. The association between outcome and independent variables was analyzed with bivariate and multivariable linear regression. P-values < 0.05 were considered statistically significant. Thematic analysis was used to analyze the qualitative data, and the findings were then referenced with the findings of the quantitative data.

**Results:**

The findings of this study showed that the sample of traditional healers found in Dessie City scored a total mean of mental health literacy of 91.81 ± 10:53. Age (*β* = -0.215, 95% CI (-0.233, -0.05), p = 0.003, informal educational status (*β* = -5.378, 95% CI (-6.505, -0.350), p = 0.029, presence of relative with a mental disorder (*β* = 6.030, 95% CI (0.073, 7.428),p = 0.046, getting information on mental illness (*β* = 6.565, 95% CI (3.432, 8.680), p = <0.001, and mental health training (*β* = 4.889, 95% CI (0.379, 6.455), p = 0.028 were variables significantly associated with mental health literacy. Traditional healers provide a variety of explanations for the causes of mental illness, including biological, psychological, and supernatural ones.

**Conclusion:**

The mean score of the Mental Health Literacy Scale (MHLS) is lower among traditional healers compared with other studies. Age, informal educational status, family history of mental illness, getting information on mental illness, and mental health training were significantly associated with mental health literacy. Therefore, different works to improve the levels of mental health literacy among traditional healers are essential.

## Background

Mental illness is a disorder that impacts cognition, emotion, and behavioural control. They significantly impair both children’s learning and adults’ ability to function in their families, places of employment, and society at large [1]. According to the World Health Organisation (WHO) report, mental disorders constitute 14% of the global burden of disease. It is estimated that 450 million individuals worldwide experience mental illness at any given time, the majority of whom reside in developing nations [2]. Additionally, the worldwide burden of disease accounts for 32.4% of years lived with disability (YLDs) and 13.0% of disability-adjusted life-years (DALYs) [3], and the overall prevalence rate of common mental disorders in Ethiopia is 22% [4].

Mental health literacy refers to the knowledge, beliefs, and attitudes about mental problems that aid their early detection, treatment, and prevention [5]. Researchers have also asserted that mental health literacy is not a singular concept but rather refers to general pre-existing belief systems’ knowledge and ideas concerning mental health illnesses. However, the majority of the literature revealed that mental health literacy among traditional healers is unfortunately low despite evidence of a high prevalence of mental illness in low and middle-income countries, including, Ethiopia [6,7]. Traditional healers respected members of the community who have some knowledge of the practice of treating mental illness with religious rites or indigenous drugs [8,9]. They have been frequently used to treat mental illness in many African and Asian nations for many years. Due to societal acceptance, they also had a successful good and a large number of clients [10]. The concurrent use of both modern and traditional mental health services in many African nations was taken as evidence of the quality of those services. It is widely accepted and practiced by all religious adherents as well as local community leaders in Ethiopia [11].

The most popular biomedical option in Ethiopia is traditional healing. The country, has only 60 psychiatrists and one mental health center, despite having approximately 100 million people. In other words, for every psychiatrist, there are 1.67 million Ethiopians [12]. Evidence shows 80% of the population rely upon traditional healers for primary health needs. This can be explained by the accessibility and affordability of traditional healers. Whilst Ethiopian health insurance often proves inadequate, traditional healers usually do not charge in advance. Instead, they are compensated with a small gift if patients indeed recover. Furthermore, traditional healers are often considered the most effective. Additionally, unlike biomedicine, it is consistent with hegemonic traditions and (religious) beliefs and is rooted in cultural and moral value systems [13]. Due to societal myths and misconceptions about mental illnesses, and the lack of resources for coordinating and integrating both modern and traditional mental health services, there is a history of stigmatization and discrimination against people with mental illnesses everywhere in the globe [14]. These actions constitute a violation of the economic, social, and cultural rights of many people, who also face restrictions on their ability to work, pursue an education, start a family, enjoy their personal liberty, exercise their right to vote, engage fully in public life, and decide for themselves how they should be treated and cared [15]. According to the 2017 Atlas report, 2,171.27 (per 100,000 populations) were disability-adjusted life years in Ethiopia [16].

Findings for the cause of mental illness reported from the previous studies were the unknown biological cause, childhood traumatic event, and traumatic event [17]. In Ethiopia, there is a widespread belief that mental disorders are caused by the evil eye, curses, stressful events, demonic possession, supernatural force, or food/drink poisoning were considered as the cause of mental illness [18]. Factors such as the socio-demographic age of respondents, marital status, socioeconomic status, living in rural areas, occupation, educational status, year of experience, social support, previous contact with the mentally ill, presence of a relative with a mental disorder, media, altered quality of life by problems with mobility, and information of mental health were significantly associated with the level of mental health literacy [5,19,20].

Currently, mental illness is growing at a fast rate in the world, and the majority of cases were treated in settings that the community preferred, such as religious and traditional institutions. Also, most of the mental health literacy studies have been conducted in Western scientific report [21,22] with not enough published research available, especially focusing on the level of mental health literacy among traditional healers regarding mental illness in our country. Therefore, the purpose of this study aimed to explore the level of mental health literacy and associated factors among traditional healers towards mental illness in Dessie City. This study will be significantly important for policymakers and different stakeholders in integrating quality mental health services that use to get better care and integrated treatment options for the mentally ill. Also, it will build on the existing information and knowledge of traditional healers. Furthermore, it will serve as baseline data for decision-makers, health planners, future researchers, and politicians.

## Method and materials

### Study setting, design, and period

A cross-sectional study was conducted using a mixed approach (qualitative and quantitative data collection methods) in Dessie City, Northeast Ethiopia, from September 1-30/2022. Dessie City administrative center is situated 401 Km Northeast of Addis Abeba in the Amhara Region of Ethiopia. It is located at 11°8′N and 39°38′E in latitude and longitude, and between 2,470 and 2,550 meters above sea level. It has a population of 350,000 people and 18 kebeles. According to data from the South Wollo Zone statistics office for the years 2016 to 2017, there were 186,571 men and 163,429 women in these populations. In the city there is one governmental comprehensive specialized hospital, eight health centers, one prison, 4 private high schools, 9 governmental high schools. The majority of residents, 58.62% of the population identified as Muslims. Orthodox Christians made up 39.92% of the population, while Protestants made up 1.15% of the total population.

#### Source of population

All traditional healers in Dessie town, North East, Ethiopia.

#### Study population

Traditional healers were available in Dessie town during the study period.

### Inclusion criteria and exclusion criteria

Traditional healers whose age’s above 18 years living in Dessie town during the time of the study were included, while traditional healers who were acutely ill, and those residents less than 6 months were excluded from the study.

### Sample size determination for quantitative

The sample size for this study was calculated using a formula for a single population mean at 95% CI, a margin of error (5%), and by taking standard deviation (SD = 10.83), and standard error (SE = 1) from the previous study conducted in the USA as there is no study so far in Ethiopia and Africa [19]. Then the sample size was calculated as follows;

$$n=\frac{{\left(Z\;\alpha {/}_{2}\sigma \right)}^{2}}{SE^{2}}=\frac{{\left(1.96\;X\;10.83\right)}^{2}}{1^{2}}=451$$

Since the total population is less than 10,000, it is finite population correction formula is used n_f_ = $\frac{n}{1+\frac{n}{N}}=\frac{451}{1+\frac{451}{1470}}$ = 345

By adding a 5% non-response rate the final sample size was 362.

### Sample size determination for qualitative

A total of 12 in-depth interviews and 3 focus group discussions were conducted to explore factors related to the level of mental health literacy. The data was collected until reaching its saturation point.

### Sampling technique for quantitative

**Fig 1:**
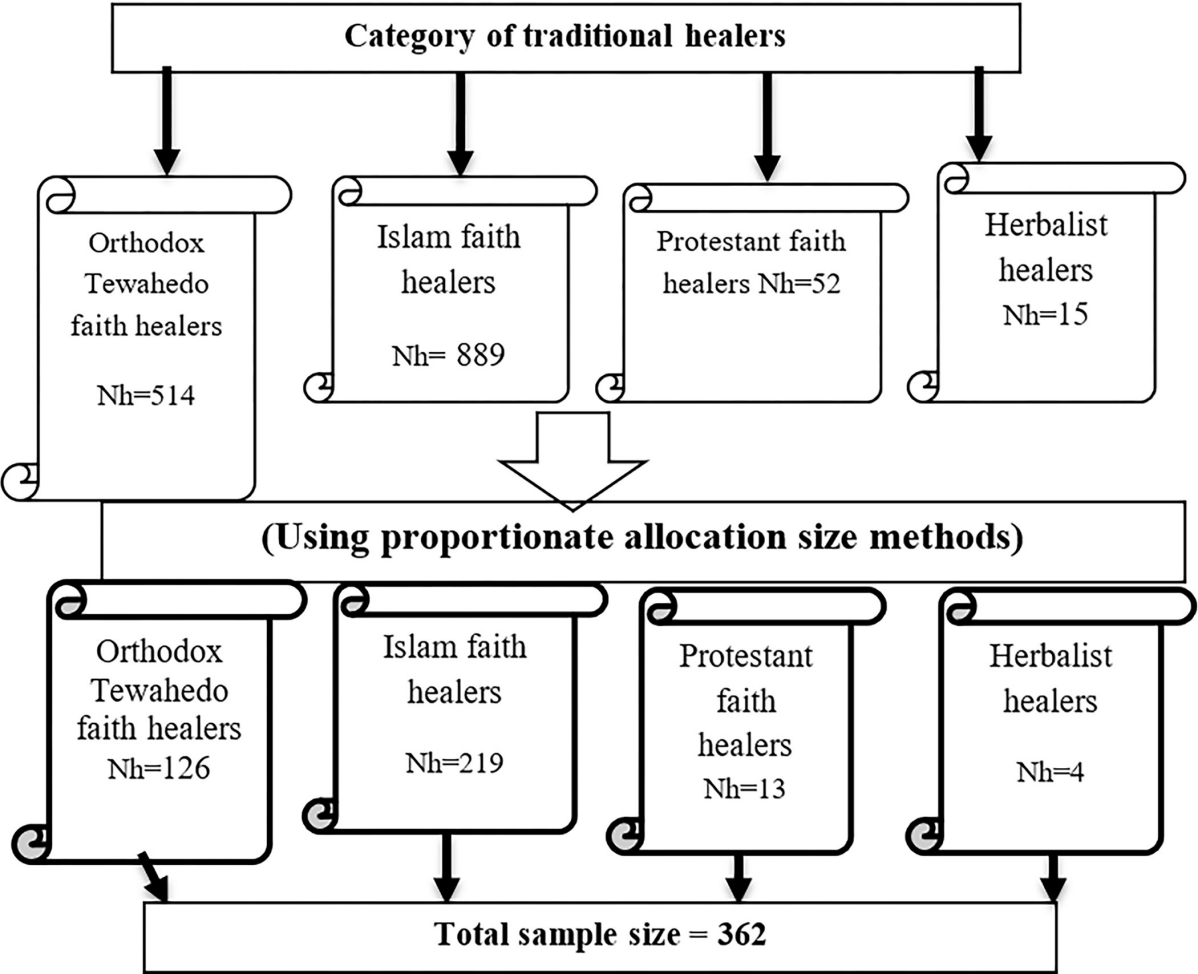
Schematic representation of sampling technique showing the number of selected samples from each category Dessie City, 2022.

The technique of how we addressed the participants is that, there was a total number of 1470 traditional healers; Orthodox Tewahedo faith healers (Nh = 514), Islam faith healers (Nh = 889), Protestant faith healers (Nh = 52), and Herbalist healers (Nh = 15) were traditional healers available in Dessie City. Information was obtained from each traditional healers head office/department. Based on the final sample size result, it was proportionally allocated to four traditional healers’ categories. There was a record list of traditional healers based on this, we selected the study unit from each group. After we found their identification number from the office coordinators’ list document, we mixed numbers on a piece of paper and then selected random numbers from mixed numbers via a lottery corresponding to a proportionally defined sample for each traditional healer’s category. The four groups of healers are common in Dessie city and accepted by the community. Most of the mentally ill patients visit them to heal. Each group of healers has different in the way they treat illness (Fig 1).

### Sampling technique for qualitative

Twelve in-depth interviews and three FGDs (each group of 8 participants) were conducted to explore the level of mental health literacy and mental health treatment practice of traditional healers. For each of the FGD study participants were purposively selected. For each in-depth interview maximum of 45 minutes to 1 hour was allocated and for each FGD maximum of 2 hours were used.

### Data collection method

#### Data collection tools for quantitative and qualitative

The Mental Health Literacy Scale (MHLS) is a 35-item questionnaire looking at the respondents’ understanding of mental health. The first 15 items are scored on a 1–4 scale with items 10, 12 & 15 being reversed scored. Items 16–35 are scored on a 1–5 scale with items 20–28 being reverse scored. The sum of all the items results in the final score (maximum score: 160; minimum score: 35). High mental health literacy was indicated by the highest score. The internal consistency (Cronbach alpha) of (MHLS) in this study was 0.79. According to an Australian study the total reliability of Cronbach a was 0.79 [5].

The Social Support Scale (The Oslo 3-items) (OSSS-3) was used to collect data regarding the strength of social support [23]. It has a score ranging from 3–14 and a higher score has indicated increasing social support. Socio-demographic factors, traditional healing-related factors, and mental health experiences were used on “yes/no” response questionnaires and were operationalized according to different works of literature. For qualitative, first, an English version of a semi-structured interview guide was prepared and then translated into the Amharic language. In order to check for consistency, it was then back-translated from English into Amharic by a professional.

### Operational definitions

**Mental health literacy:** The ability to recognize mental disorders; knowing about source of information, beliefs about and known causes of mental illness, and treatment strategies available for mental illness. Using MHLS, the scale ranged from 35–160 and the increasing score indicated an increasing level of mental health literacy [5].

**Traditional healers**: Herbalists and faith healers (Imam/Sheik, orthodox church clergy, protestant pastor) found in Dessie City [24].

### Data collection procedures

#### For quantitative study

The questionnaire was initially prepared in English, then translated into the Amharic language, and finally back into English to ensure uniformity. Additionally, we use standard tools to determine the outcome variable by giving two days of training for data collectors and supervisors. The data was collected through face-to-face interviews with pre-tested interviewer-administered questionnaires. Five data collectors (psychiatry nursing) were employed for one month of data collection periods and supervised by one supervisor. Then, data was collected from all available traditional healers in Dessie Town. In Kombolcha town, 5% (n = 18) of the participants took part in the pre-test, which aimed to identify any potential issues with the data collection methods and suggest changes to the questionnaire. The principal investigator and supervisor regularly oversaw and assisted the data collectors. Supervisors and principal investigators checked the data daily for consistency and completeness during the data collection period.

#### For qualitative study

The in depth interview guiding and checklist was prepared in English and the it was translated into Amharic and back to English to ensure the reliability of information. Pretest was was done to test the tools, trustworthiness (like credibility, dependability, confirmability and transferability), reliability and interview location, audio recording sound and time frame before the main study was conducted. For qualitative data collection, the IDI and FGD guide was employed, and discussions were anticipated to explore their levels of mental health literacy and the primary source of information, what participants perceive about the causes and manifestations of mental illness, what they feel about the illness and its effects, whether there is modern medication for mental illness, and what the preferred place of treatment for the patients with mental illness and its relation with family history and integrate traditional healing with the modern healthcare. A peer review and member checking were used to ensure the credibility and trustworthiness of the study. Member checking was carried out during the interviewing process by restating or summarizing the responses of participants to validate their responses. Three data collectors with the investigators’ supervision collected both the twelve in-depth interviews and three FGDs (each group of 8 participants) were conducted. For each in-depth interview maximum of 45 minutes to 1 hour was allocated and for each FGD maximum of 2 hours were used.

### Data processing, and analysis

#### For quantitative study

Data was coded and entered into EPI data version 4.6 and then exported to SPSS Version 26.0 for analysis. Descriptive statistics such as texts, frequency, percentage, tables for categorical data, and mean, median, and standard deviation for continuous variables were used. Correlation analysis was used to determine the relationship between mental health literacy and independent variables. All variables were entered into bivariate linear regression to identify associated factors of mental health literacy and variables with P-value < 0.25 was considered as candidates for multivariable linear regression analysis. In multivariate linear regression analysis variables with a p-value < 0.05 was considered as statistically significant. Before performing the linear regression analysis, the following assumptions of linear regression were checked: normality was checked using the normal histogram curve and the Kolmogorov-Smirnov test; linearity was checked using the (quantile-quantile) QQ plot and histogram; no outlier was found during the outlier test; multicollinearity was checked using the variance inflation factor (VIF), and all variables had a VIF <2; homoscedasticity was verified using the Levene’s test in which all variables that were *P* > 0:05 indicate no heteroskedasticity. The independent observation was checked using the Durbin-Watson value, and the result had a value of 1.97.

#### For qualitative study

After collecting the data through the interviews the next step was transcribing all the audio recordings of interviews directly into Amharic languages which translated to the English language. Then the data was coded and based on the similarity and relatedness of the coded data different themes were developed [25]. A principal investigator carried out the initial coding utilizing inductive methods [26]. Finally, the analysis included code categorization and it was analyzed using sub-them manually once the first coding was completed and, the finding was triangulated with the quantitative finding for drawing conclusions from the study.

### Ethical consideration

Ethical clearance was obtained and approved by the Institutional Review Board (IRB) of Wollo University College of Medicine and Health Sciences with IRB *RCSPG-206/14*. Submission of the ethical approval letter of the board was given to all concerned bodies. All study participants were told that participation was completely voluntary, that written informed consent was obtained, and that they could withdraw from the study at any if they were not comfortable with the questionnaire. Confidentiality was assured to participants and the participants were not being expected to write their names at the time of responding to the questions. All methods were performed in accordance with the relevant guidelines and regulations.

## Results

### Quantitative result

#### Sociodemographic characteristics of respondents

A total of 343 respondents were involved in this study and the response rate was 94.75%. The mean ± SD age of respondents was 44.38 ± 12.10, which ranged from 25 to 68 years. The majority of the participants, 212 (61.8%), 277(80.8%), and 293(85.4%) were Muslim, married, and Amhara respectively. According to the participant’s responses, 134(39.1%) were informal education and 302(88.0%) were private jobs in their occupation. Most of the participants, 209 (60.9%) were Sheik/Imam followed by 120 (35.0%) orthodox priests. The median income of the respondents was 5000 with an interquartile range of 4000 ETB. Regarding traditional healing, the majority of the participants 276 (80.5%) were trained informally (Table 1).

**Table 1:** Socio-demographic characteristics of traditional healers in Dessie, Northeast, Ethiopia, 2022 (N = 343).

Variables	Category	Frequency	Percentage (%)
Faith	Islam	212	61.8
	Orthodox	121	35.3
	Protestant	10	2.9
Marital status	Has Spouse	277	80.8
	No Spouse	66	19.2
Ethnicity	Amhara	293	85.4
	Tigre	37	10.8
	Other*	13	3.8
Educational qualification	No formal education	134	39.1
	Primary	76	22.2
	Secondary	71	20.7
	College & above	62	18.1
Occupation	Private Worker	302	88.0
	Merchant	41	12.0
Traditional healer type	Imam/Sheik	209	60.9
	EOTC priest	120	35.0
	Pastor	10	2.9
	Herbalists	4	1.2
Type of training on traditional healing	Formal	67	19.5
	Informal	276	80.5

Others*: South, Oromo.

### Mental health experiences and social support of participants

The mean social support of the respondents using the Oslo social support scale (OSSS) was 8.5359 (SD = ± 2.559), with a minimum and maximum score of 5 and 14 respectively. According to this finding, 54 (15.7%) of respondents had received mental illness training. More than two third of the participants 228(66.5%) knew any person diagnosed with mental illness other than a family member while 27(7.9%) of the respondents had suffered from mental illness. Among participants, 32 (9.3%) respondents with a presence of a relative with a mental disorder, and 278 (81%) participants got information on mental illness through different media. From the respondents, 61(17.8%) of the traditional healers had received a history of seeking help from health professionals in treating mental illness. Among the participants, 51(14.9%) had reported a history of treating mental illness (Table 2).

**Table 2:** Mental health experiences of participants of traditional healers in Dessie, Northeast, Ethiopia, 2022 (N = 343).

Variables	Category	Frequency	Percentage (%)
Training on mental illness received	Yes	54	15.7
	No	289	84.3
Suffered from any mental illness	Yes	27	7.9
	No	316	92.1
Presence of a relative with a mental disorder	Yes	32	9.3
	No	311	90.7
Knowing any person diagnosed with mental illness	Yes	228	66.5
	No	115	33.5
Get information on mental illness	Yes	278	81.0
	No	65	19.0
History of seeking help health professionals in treating mental illness	Yes	61	17.8
	No	282	82.2
History of treating mental illness	Yes	51	14.9
	No	292	85.1
Willing to work in collaboration with health workers	Yes	265	77.3
	No	78	22.7

More than three fourth 265(77.3%) are willing to work jointly in collaboration with mental health workers, which is also supported by qualitatively…..*I am delighted to cooperate with medical institutions since collaboration is a very significant factor in providing service users with appropriate management*, *which lessens the burden of illness*.

### Mental health literacy among traditional healers

The mean total mental health literacy score among respondents was found to be 91.81 ± 10:53. According to a qualitative finding, the majority of the participants in this study use the colloquial term “ebdet,” which implies “madness.” *Mad people were weak-willed and indifferent individuals who were detrimental to healthy communities*. *They wandered the streets*, *yelled and spoke loudly*, *frightened or fought with others*, *ran barefoot*, *and slept in the trash*.

Additionally, on causes of mental illness, there are groups of traditional healers who think that bad spirits or God’s punishment are the most commonly reported. “45-year male participant*…Mental illness may result from a person’s sin or occasionally from God’s desire to teach him something crucial about his personal*, *social*, *and spiritual life*”. Additionally, *“*50-year FGD male participant*…When those with evil power exercise lethal power over others by their eye*, *this is known as the “evil eye” or “Buda*,*” and it is thought to be a contributing factor in mental illness”*. Another report, *“*39 Male ID participant*…Demons or bad spirits known as Zar/Jinni sprites have the ability to disrupt human life and have their own identities*. *However*, *when we pray using the Holy Bible on the possessed person*, *they are exorcised and the sufferer is freed”*. Also, *“*43 Male ID participant*…Dgimt/Debteras*, *which is an evil work of some people on others due to someone being envious of a friend*, *a neighbor*, *or anyone else they know that poses a harmful effect on the victim*. *Jealousy can be linked to academic success*, *professional success*, *or social success”*. Likewise, *“*39 Male ID participant…*stated that stress*, *financial difficulties*, *a lack of social support*, *unemployment*, *conflicts within the family*, *the death of loved ones and alcohol (like Tela*, *and Areque) and drug addiction*, *including khat*, *and cigarette smoking can lead to mental illness*.*“I remember a young man who had a significant alcohol intake*, *manifested behavioral changes*, *and frequently bothered his family and neighbors*. *Later*, *as a result of this he developed poor self-care*, *he fled the family house and started shouting as people passed by on the street*. *He usually smokes cigarettes and chaws khat*. *This might be the cause of his illness”*.

In the history of seeking help from health professionals for treating mental illness, most of their views on the understanding of self-help and professional help were centered on indigenous and religious knowledge, although some of them did have some awareness of professional support. “56 male ID participants… *God holds our lives in his hands*, *so if we sincerely prayed to him*, *he might make us well and free from any illness*. *Therefore*, *in my opinion*, *a person can help himself by praying to God”*. In addition, *“*37 male ID participants… *My father taught me how to prepare various medications*, *so I know how to help myself*. *Even I have access to a variety of medications that lower stress and guard against mental health issues”*. Also, “31 male IDI participants… *I do believe in spiritual healing*, *yet most patients with mental illnesses are better treated in a medical facility*. *Because I am aware of one woman who was a mentally ill patient restrained and experienced a remarkable shift while taking medication from the hospital”*.

The traditional healers also brought up a significant issue regarding the sources they turn to for information on mental illness. Most of them stated that they prefer to consult different religious texts and more experienced healers. *“*36 Male IDI participant*…I resembling to seek the most experienced dads or study various holy books to get information about any illnesses”*.

However, the attitudes of the healers towards encouraging acceptance and acceptable-seeking actions is vary. *“*36 Male IDI participant*…God has already given me the wisdom to heal various people*. *If I have a mental illness*, *I will go to the hospital and get medical assistance in addition to praying and using holy water*. In another way, *“*53 Male IDI participant*… God is almighty*, *and this is where we can find all the answers to our difficulties*. *Therefore*, *in my opinion*, *fervently requesting God’s assistance via heartfelt prayer can resolve any type of health issue*.

#### Factors associated with mental health literacy among traditional healers

Bivariate and multivariable linear regression analysis was done to identify factors associated with mental health literacy among traditional healers. In the bivariate analysis, income, age, marital status, educational status, occupation, social support, presence of a relative with a mental disorder, training on traditional healing, get information on mental illness, mental health training, and history of seeking help from health professional showed a p-value of <0.25 and became candidates for multivariable analysis.

In multivariable linear logistic regression, variables; age, educational status, presence of a relative with a mental disorder, getting information on mental illness, and mental health training were found to be statistically associated with mental health literacy at a p-value less than 0.05. Accordingly, age (*β* = -0.215, 95% CI (-0.233, -0.05), *P* = 0:003), no formal educational status (*β* = -5.378, 95% CI (-6.505, -0.350), *P* = 0:029), presence of relative with a mental disorder (*β* = 6.030, 95% CI (0.073, 7.428), *P* = 0.046), get information on mental illness (*β* = 6.565, 95% CI (3.432, 8.680), *P*<0:001), and mental health training (*β* = 4.889, 95% CI (0.379, 6.455), *P* = 0:028) were variables significantly associated with mental health literacy. These factors contributed to 72.5% of the variation in mental health literacy among traditional healers (r = 0. 86, r2 = 0.732, f = 31.88 p<0.001) This was interpreted as a unit increase in the age of the participants; the mental health literacy score decreased by 0.215 units (*P* < 0:003). Having an informal educational status decreases the mental health literacy score by 5.378 (*P* < 0:029) as compared with college and above. The presence of a relative with a mental disorder increases the mental health literacy score by 6.030 (*P* = 0.046) as compared with those who have an absence relative with a mental disorder. Also, participants who got information on mental illness were more likely to have a higher level of mental health literacy by 6.565 (*P* = *P*<0:001) times than not getting information on mental illness. Moreover, this study found that participants who took mental health training increases their ***mental*** health literacy score by 4.889 (*P* = 0:028) (Table 3).

**Table 3:** Linear regression analysis of respondents in Dessie City, Northeast, Ethiopia, 2022 (N = 343).

Variables	Category	Simple linear regression		Multiple linear regression		95% CI	
		*Unstandardized* *coefficients*		*Unstandardized* *coefficients*		Lower	Upper
		*B*	*Sig*	*B*	*Sig*		
Income		0.000	0.238	0.000	0.144	0.000	0.001
Age		-0.215	0.000	-0.141	**0.003**	-0.233	-0.050
Marital status	Spouse	1.833	0.205	1.796	0.178	-0.823	4.414
	No spouse*						
Educational status	Informal	-5.378	0.001	-3.427	**0.029**	-6.505	-0.350
	Primary	1.370	0.431	1.447	0.389	-1.850	4.745
	Secondary	0.224	0.899	0.174	0.919	-3.176	3.524
	College &above*						
Occupation	Private	2.032	0.247	-0.496	0.765	-3.756	2.765
	Merchant*						
Social support		0.300	0.179	0.211	0.305	-0.193	0.616
Presence of relative with a mental disorder	Yes	6.030	0.002	3.750	**0.046**	0.073	7.428
	No*						
Training on traditional heal	Formal*	4.331	0.002	1.328	0.395	-1.741	4.397
	Informal						
Get information on mental illness	Yes	6.565	0.000	6.056	**<0.001**	3.432	8.680
	No*						
Mental health training	Yes	4.889	0.002	3.417	**0.028**	0.379	6.455
	No*						
History of seeking help from health professionals	Yes	2.164	0.191	0.736	0.669	-2.652	4.124
	No*						

Key:- *:-Reference, significant in multi-linear regression at p-value <0.05).

## Discussion

This study aimed at estimating the levels of mental health literacy among traditional healers in Dessie City and identifying its correlates. The overall mean score of participants’ mental health literacy score was 91.81 ± 10:53. In our study, the overall mean score of mental health literacy was lower score than those conducted in the USA (134.20 ± 10:83) [19], Iranian community (113.54± 10.34) [27], and Australia 127.38 (±) [5]. The possible reasons might be due to the better access to health care providers and their higher levels of education, which helps easily recognize and understand mental health conditions from various perspectives in the USA and Australia [5,19]. This implies the collaboration between healers and health professionals may create an opportunity for healers to share their understanding of many aspects of mental health with other professionals. Additionally, fewer mental health specialists are available, and this lack of information sources has a direct bearing on the lower mental health literacy in Ethiopia.

Regarding the associated factors, in this study age was negatively associated with mental health literacy score (*β* = -0.215, 95% CI (-0.233, -0.05), *P* = 0:003). This means as age increases by a unit the mental health literacy score decreased by 0.215 units. The results of this study were consistent with the results of other studies conducted in China [28]. This could be due to differences in socio-political environments that different generations and education have experienced. This study found that informal educational status, as compared with College and above, decreases mental health literacy score by 5.378 units (*β* = -5.378, 95% CI (-6.505, 0.350), *P* = 0:029). The results of various studies done in China [28] and Malaysia [29] revealed that education level and MHL had a substantial association and as educational level increases, the level of MHL likewise improves. Also, considerably improved with higher levels of education is the capacity to identify mental problems [30].

This study found that the presence of a relative with a mental disorder increases the mental health literacy score by 6.030 units (*β* = 6.030, 95% CI (0.073, 7.428), *P* = 0.046), as compared with those who had absence relative with a mental disorder. The results of this study were supported by the result of other study done in Singapore [31]. The possible explanation for this finding is individuals with the presence of a relative with a mental disorder have a chance to increase be more familiar to recognize the manifestation which improves the patients’ social support and encourages patients to seek professional help, this contributes to increased mental health literacy scores. Also, a qualitative study supported this, *“32 Male ID participants said that… due to my mother’s health situation*, *I become familiar with several mental illness symptoms*, *which has made me more aware of the value of receiving modern therapy and knowing factors that worsen his illness and things that help to improvement”*.

Based on the results of this study, getting information on mental illness increases the mental health score by 6.565 units than not getting information on mental illness (*β* = 6.565, 95% CI (3.432, 8.680), *P*<0:001). According to the study’s findings, mental health literacy and access to information about mental illness are significantly correlated. Those who obtained more information in this area improved people’s capacity to identify mental illnesses [30]. The results of this study were supported by various study findings [28,31]. According to the findings of a study by *Gulliver*, one of the main causes of low MHL is the failure to seek out information on mental problems, which makes it difficult to identify mental diseases and less likely to seek out mental health care [28]. The qualitative finding revealed by the participant supports those who get information on mental illness increase their mental health score. *“56 Male ID participants…I did not have a lot of information about mental illness before*, *but when I was watching a television program that had guests who talked about health-related conditions and that gave me some understanding of mental health”*. This is a comparison with the previous study done by Jorm et al. in Australia, individuals with better recognition of schizophrenia and depression were more likely to receive a variety of mental health services, such as counseling from mental health professionals, medications, psychotherapy, and psychiatric admissions[32].

Finally, this study found that with mental health training increases, the mental health literacy score increases by 4.889 (*β* = 4.889, 95% CI (0.379, 6.455), *P* = 0:028), which was a similar result to the study done in the USA [19] and Kenya [33]. This important correlation might be caused by the direct, powerful impact of mental health education on improving the understanding of mental disorders. This is supported by participants qualitatively*“32-year Male FGD participants…We learned about mental illness in certain religious courses in college*, *and this kind of education is highly beneficial to improve understanding”*. In developing nations employing a communication strategy aimed at publicly explaining and promoting an integrated mental system, informing and motivating biomedical staff, and encouraging and training healers. Traditional healers can better integrate within the ever-evolving healthcare systems that are relying more heavily on patient-centered, holistic approaches to integrating mental and physical healthcare by collaborating with biomedical resources [34].

### Strength and limitations of the study

This study’s strength is that it explores the factors from both quantitative and qualitative perspectives to examine them in many dimensions, but it has some limitations. First, the data was collected based on retrospective self-report and it could be biased due to social desirability and recall. Secondly, because the study was cross-sectional, the cause-and-effect link between the variables could not be examined. Finally, the participants of the study were recruited from only found in Dessie city, and restricted to presenting an enormous amount of information on the levels of mental health literacy among the different populations of the Ethiopian traditional healers. It is suggested that in future studies, the status of MHL be investigated across different Ethiopian traditional healers.

### Conclusion and recommendation

The mean score of the Mental Health Literacy Scale (MHLS) is lower among traditional healers compared with other studies. Age, informal educational status, family history of mental, get information on mental illness, and mental health training were significantly associated with mental health literacy. In Ethiopia, all sociodemographic groups are experiencing an increase in mental health problems, however, there are currently insufficient efforts to raise awareness among the healers, various interventions can be considered including the development of short mental health training for healers are essential. Social media and internet-based resources can also be used to disseminate further information on various mental health conditions. Also, future research should concentrate on the best methods for educating healers about mental health.

## Supporting information

S1 File (XLS)

## Abbreviations

CI: Confidence Interval
ETB: Ethiopian Birr
FGD: Focus Group Discussion
G.C: Gregorian calenda
IDI: In-Depth Interview
MHL: Mental Health Literac
MHLQ: Mental Health Literacy questionnaire
OSSS-3: Oslo Social Support Scale
SD: Standard Deviation
SPSS: Statically Package for Social Science
USA: United States of America
WHO: World Mental Health
